# Pneumococcal Polysaccharide Vaccine Ameliorates Murine Lupus

**DOI:** 10.3389/fimmu.2019.02695

**Published:** 2019-11-20

**Authors:** Chiara Cantarelli, Chiara Guglielmo, Susan Hartzell, Fadi El Salem, Sofia Andrighetto, Victor P. Gazivoda, Enrico Fiaccadori, Gaetano La Manna, Gianluigi Zaza, Jeremy Leventhal, Ioannis Tassiulas, Paolo Cravedi

**Affiliations:** ^1^Division of Nephrology, Department of Medicine, Icahn School of Medicine at Mount Sinai, New York, NY, United States; ^2^Unità Operativa Complessa di Nefrologia, Azienda Ospedaliera-Universitaria Parma, Dipartimento di Medicina e Chirurgia, Università di Parma, Parma, Italy; ^3^Department of Experimental Diagnostic and Specialty Medicine (DIMES), Nephrology, Dialysis and Renal Transplant Unit, St. Orsola Hospital, University of Bologna, Bologna, Italy; ^4^Department of Pathology, Icahn School of Medicine at Mount Sinai, New York, NY, United States; ^5^Renal Unit, Department of Medicine, University-Hospital of Verona, Verona, Italy; ^6^Department of Surgery, Maimonides Medical Center, New York, NY, United States; ^7^Division of Rheumatology, Department of Medicine, Icahn School of Medicine at Mount Sinai, New York, NY, United States

**Keywords:** lupus, prevnar, vaccination, T_**FH**_, T_**FR**_

## Abstract

Current guidelines encourage administering pneumococcal vaccine Prevnar-13 to patients with lupus, but whether such vaccinations affect disease severity is unclear. To address this issue, we treated 3-month-old MRL-*lpr* mice, that spontaneously develop a lupus-like syndrome, with Prevnar-13 or vehicle control. After 3 months, we quantified circulating anti-Pneumococcal polysaccharide capsule (PPS) antibodies and signs of disease severity, including albuminuria, renal histology and skin severity score. We also compared immunophenotypes and function of T and B cells from treated and untreated animals. Prevnar-13 elicited the formation of anti-pneumococcal IgM and IgG. Prevnar-13 treated animals showed reduced albuminuria, renal histological lesions, and milder dermatitis compared to vehicle-treated controls. Mitigated disease severity was associated with reduced and increased T follicular helper cells (T_FH_) and T follicular regulatory cells (T_FR_), respectively, in Prevnar-treated animals. T cells from Prevnar-13 vaccinated mice showed differential cytokine production after aCD3/aCD28 stimulation, with significantly decreased IL-17 and IL-4, and increased IL-10 production compared to non-vaccinated mice. In conclusion, pneumococcal vaccination elicits anti-pneumococcal antibody response and ameliorates disease severity in MRL-*lpr* mice, which associates with fewer T_FH_ and increased T_FR_. Together, the data support use of Prevnar vaccination in individuals with SLE.

## Introduction

Systemic lupus erythematosus (SLE) is the prototypical systemic autoimmune disease with complex genetic and immune system pathogenic mechanisms operative throughout the disease course (1, 2). While several therapeutic targets have been identified, treatment of the most severe manifestations (e.g., lupus nephritis) consists of glucocorticoids and immunosuppressive/cytotoxic agents that have significant side effects and risks (3–5). Long-term use of immunosuppressive medications is a major risk factor for infection-related morbidity and mortality in SLE patients (6, 7). Bacteria are the most common pathogens seen, with *Streptococcus pneumoniae* being the most frequent microorganism involved in respiratory tract infections (8–11). Accordingly, international guidelines recommend pneumococcal vaccine administration to the majority of patients with SLE (12).

Despite recommendations, vaccination rates in patients with SLE are only 50–60% (13–15). Initial concerns centered on questions of vaccine efficacy, but numerous studies over the past 30 years confirm that vaccinations against *Influenza* and *Pneumococcus* are equally efficacious in patients with SLE (16). Pneumococcal conjugate vaccine (PCV; Prevnar-13) contains polysaccharides from the 13 most common pathological pneumococcal serotypes and is recommended for immunosuppressed individuals, including patients with SLE. Additional concerns that anti-pneumococcal vaccination might worsen disease severity have proven obstructive to enacting guideline recommendations. Although a study of 24 SLE patients showed no significant change in autoantibody titers or C3/C4 serum levels 2 months after anti-pneumococcal vaccination (17), onset of SLE has been described after immunization against tetanus (18, 19), hepatitis B (18, 20), and other vaccines (21), providing cause for concern.

Several mechanisms have been proposed to account for induction of post-vaccination autoantibody-mediated disease. Molecular mimicry of host structures by pathogens is a well-known theory offering an explanation for initial activation of autoreactive B cells that does not require autoantigen priming. Furthermore, it was demonstrated that normal post-vaccination anti-pneumococcal antibody responses in healthy individuals can express lupus-associated anti-DNA idiotypes (22). Similarly, a high percentage of anti-bacterial antibodies against *Streptococcus pneumoniae* produced in patients with lupus are capable of binding double-stranded DNA (23). These findings are cause for concern despite official recommendations.

Conversely, anti-pneumococcal vaccine may also exert anti-inflammatory effect. While polysaccharides in Prevnar vaccine elicit T cell independent B cell responses, the vaccine is conjugated to *Diphtheria* CRM197 protein, producing T cell dependent responses, as well (24, 25). In a murine model of allergic rhinitis and allergic airway disease it was shown that conjugated pneumococcal vaccine regulated allergen-specific T_H_2 responses and induced regulatory T cells (T_REG_) (26). Similarly capsular polysaccharide A (PSA) from *Bacteroides fragilis* was shown to play an important role in regulating demyelination in experimental allergic encephalomyelitis, an animal model of multiple sclerosis (27).

Despite justified concerns preventing guideline enaction, mechanistic studies investigating the effect of Prevnar-13 on immune function in SLE are lacking. To address this issue, herein we report effects of Prevnar-13 on immunological and clinical parameters of the lupus-like disease occurring in MRL-*lpr* mice.

## Materials and Methods

### Mice

MRL-Fas^lpr^ (MRL-*lpr*) mice were purchased from The Jackson Laboratory (Bar Harbor, ME). Animals were housed at Icahn School of Medicine at Mount Sinai (New York, NY). Study protocols were approved by the Institutional Animal Care and Use Committee at Icahn School of Medicine at Mount Sinai.

### Procedures

Twelve-week-old MRL-*lpr* male mice were injected with vehicle (PBS) or Prevnar-13 (pneumococcal 13-valent conjugate vaccine injection, suspension, Wyeth LLC, Pfizer Inc, NY) in a single dose of 50 μl/per mouse, i.p., and sacrificed after 12 weeks. In selected experiment, mice were also given mycophenolate mofetil (MMF; 0.563%) added to standard chow (28) for the entire duration of the experiment (months of age 3–6).

### Pneumococcal Specific Indirect ELISA

Peripheral blood was collected through retro-orbital blood draw before vaccination with Prevnar, and after sacrifice by cardiac puncture. Serum was then isolated from the peripheral blood and analyzed for anti-PPS IgM and IgG antibodies. Serum was diluted to 1:100 in PBS and transferred to 96 well ELISA plates coated with 10 μg/mL per well of polysaccharides. ELISA was then carried out according to the procedure described in the WHO guidelines for PPS ELISA (https://www.vaccine.uab.edu/uploads/mdocs/ELISAProtocol(007sp).pdf) with some modifications. The goat anti-murine IgM antibody used to detect PPS IgM by custom indirect ELISA was purchased from Jackson ImmunoResearch (115–006-020; West Grove, PA). The anti-murine IgG1 kappa antibody used to detect PPS IgG by custom indirect ELISA was purchased from Sigma-Aldrich (M7894; St. Louis, MO).

### Assessment of Dermatitis

MRL-*lpr* mice develop inflammatory skin lesions on the forehead, ears and dorsum of the neck, which were scored on a scale of 0–3, where 0 = no visible skin changes, 1 = minimal hair loss with redness and a few scattered lesions, 2 = redness, scabbing, and hair loss with a small area of involvement, and 3 = ulcerations with an extensive area of involvement as described previously (29).

### Renal Histology

After confirmation of stage 3 anesthesia, mice were transcardially perfused with periodate-lysin-paraformaldehyde fixate at 4% in phosphate buffered saline (PBS), and the kidneys were dissected out. Kidney samples were either frozen in Optimal Cutting Temperature compound (Tissue Tek O.C.T., Sakura, CA) or fixed in 10% formalin and then embedded in paraffin.

### Light Microscopy

Mice kidneys were cut and stained with Periodic acid-Schiff (PAS) staining and subsequently were evaluated by a renal pathologist. Lesions were scored according to the National Institutes of Health (NIH) activity and chronicity index adapted by the International Society of Nephrology/Renal Pathology Society classification for lupus nephritis (30). Scoring system is based on glomerular histopathological changes. The scoring criteria include these findings: endocapillary hypercellularity, neutrophils/karyorrhexis, fibrinoid necrosis, wire loop lesions and/or hyaline thrombi, cellular/fibrocellular crescents, and interstitial inflammation. A scale from 0 to 3 is used corresponding to how many glomeruli are affected, in which no lesion = 0, < 25% of glomeruli = 1, 25% – 50% of glomeruli = 2, or > 50% of glomeruli = 3.

### Immunofluorescence Analysis in Renal Tissue

Samples were frozen in O.C.T. compound. For staining, 5 μm thick cryosections were incubated with PBS for 5 min then with blocking solution containing PBS, 2% bovine serum albumin, 2% fetal bovine serum, and 0.2% fish gelatin for 30 min at room temperature (RT). For renal tissue markers, the following antibodies were used: rat anti-mouse C3b (1:50; Hycult Biotech, Wayne, PA) incubation at 4°C overnight followed by indirect staining with secondary antibody (goat anti-rat Alexa fluor - 488) 1 h at RT; or rat anti-mouse IgG-FITC antibody (1:50; eBioscience, San Diego, CA) incubation at 4°C overnight. At least 65 glomeruli/section for each animal were randomly acquired using fluorescence non-confocal laser scanning microscopy (Zeiss AxioImager Z2M with ApoTome.2). Surface antibody expression was estimated by constructing a contour mask on the merged image. Software ImageJ was used to quantify C3b and IgG staining intensity.

### Urine Albumin/Creatinine

Urine creatinine was quantified using commercial kits from Cayman Chemical (Ann Arbor, MI). Urine albumin was determined using a commercial assay from Bethyl Laboratory Inc (Houston, TX). Urine albumin excretion was expressed as the ratio of urine albumin to creatinine (A/C).

### Splenic T and B Lymphocyte Cytokines

Single cell suspensions of splenic lymphocytes were separated using EasySep negative selection kits from Stem Cell Technologies (Vancouver, BC): T cell kits enriched CD3^+^ cells and B cell kits enriched CD19^+^ cells. CD3^+^ T cells were stimulated with Dynabeads Mouse T-activator CD3/CD28 (Invitrogen) in a 1:1 bead-to-cell ratio, and isolated CD19^+^ B cells were stimulated with 1 μg/mL of TLR7/8 agonist R848 (InvivoGen) or 20 ng/mL of LPS-EB (InvivoGen, San Diego, CA). Supernatants were collected after 1 day for T cells and after 5 days for B cells. Cytokine quantification of IL-4 (R&D Systems, MN), IFN-γ (BD Biosciences, CA), IL-17A (eBioscience), IL-10 (BD Biosciences), TNF-α (BD Biosciences), and IL-6 (R&D Systems) were carried out by sandwich ELISA per manufacturer's instructions.

### Flow Cytometry

We used standard approaches for surface and intracellular staining as published (31). For surface staining, we used PE-Cy7 and BV510-anti-CD4 (clone RM4–5 and GK1.5 respectively), PE-Cy7-anti-CD8 (clone RPA-T8); FITC-anti-CD25 (clone 15F9); biotinylated anti-CXCR5 followed by Pacific Blue streptavidin, PerCP-Cy5.5-anti-TCRb (clone H57–597), PE-anti-ICOS (clone 15F9), PE-Cy7-anti-PD-1 (clone RMP1–30), PerCP-Cy 5.5-anti-B220 (clone RA 3–6 B2), APC-Cy7-anti-IgD (clone 11–26c), PE-Cy7-anti-IgM (clone eB121–15F9), PE-anti-FAS (clone Jo2), FITC-anti-GL7 (clone Ly-77). For intracellular staining, we used APC and FITC-anti-FoxP3 (clones MF23 and FJK-16S). All these antibodies were obtained from BD Pharmingen.

Data were acquired (10,000 to 100,000 events) on a three-laser Canto II flow cytometer (BD Biosciences) and analyzed using FlowJo (https://www.flowjo.com) software.

### Statistical Analyses

Comparisons of continuous variables between two groups were analyzed by *t*-tests. For histological score comparisons, we used Wilcoxon test. Two-way repeated measures ANOVA test was used for multiple comparisons among treatment groups. *P*-values < 0.05 were considered significant. All statistical analyses were performed using GraphPad Prism (version 8 for Windows, GraphPad Software, Inc.).

## Results

### Prevnar-13 Vaccination Promotes an Anti-Pneumococcal IgG Response in MRL-*lpr* Mice

**Figure 1:**
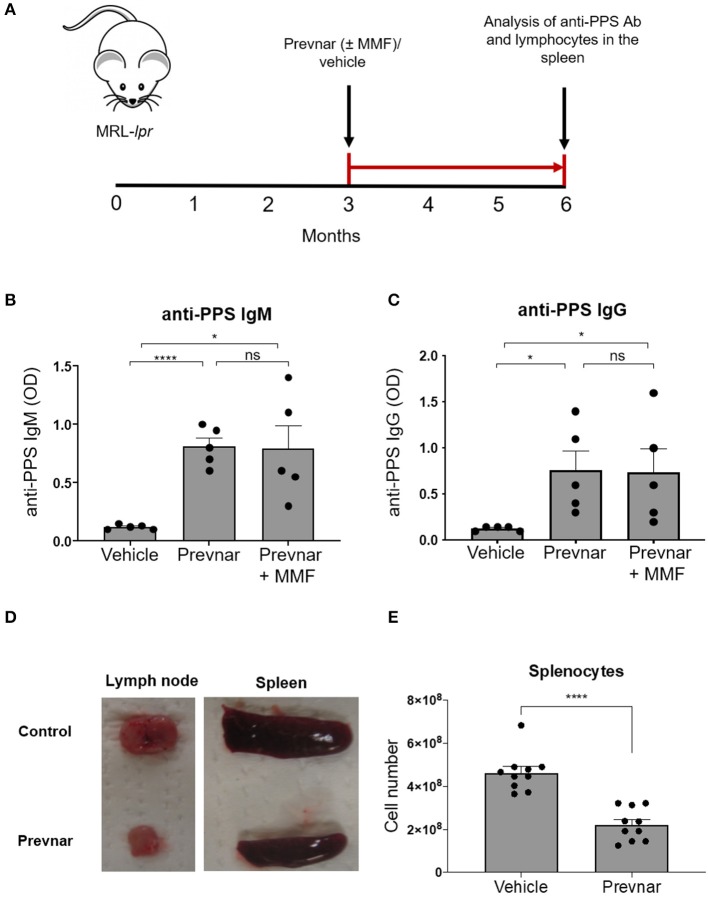
Prevnar-13 vaccination promotes an anti-pneumococcal IgG response and reduces lymphocyte numbers in MRL-*lpr* mice. Twelve-week-old MRL-*lpr* mice were given intraperitoneal injection with Prevnar-13 or vehicle control **(A)** with or without mycophenolate mofetil (MMF). Twelve weeks later, we measured serum anti-pneumococcal polysaccharide (anti-PPS) IgM **(B)** and IgG **(C)** levels. In a separate set of experiments, we treated animals with Prevnar or vehicle (no MMF) and we sacrificed them for quantification of disease severity and immunological studies. Representative pictures of lymph nodes and spleens **(D)** and splenocyte number **(E)** in Prevnar-13 or vehicle-treated MRL-*lpr* mice. Data are presented as mean ± SEM. They represent a total of 5 mice per group **(B,C)** or 10 mice per group **(D,E)** obtained over two or three independent experiment, respectively; *p < 0.05, ****p < 0.0001, ns, not significant by unpaired *t-*test.

SLE, and medications used to treat it, may prevent effective vaccination responses. To test this, we injected 12-week-old MRL-*lpr* mice with Prevnar-13 or PBS as control. We quantified anti-PPS specific IgM and IgG antibodies 12 weeks later (Figure 1A). Anti-PPS IgM and IgG antibody responses were detected in all vaccinated mice (Figures 1B,C) while none were seen in control mice. To determine how immunosuppression affects antibody responses in SLE, we vaccinated MRL-*lpr* mice receiving mycophenolate mofetil (MMF), a commonly used treatment in human SLE. PPS-specific antibody levels in MMF treated mice were similar to those in mice receiving vaccine alone and significantly higher than in unvaccinated controls (Figures 1B,C). Together, these data show that Prevnar-13 vaccination elicits an anti-pneumococcal IgM and IgG response in MRL-*lpr* mice even under MMF immunosuppressive therapy.

### Prevnar-13 Vaccination Reduces Lymphadenopathy in MRL-*lpr* Mice

We next determined whether Prevnar-13 vaccination affects autoimmune disease in lymph nodes, spleen and skin of MRL-*lpr* mice. Similar to many SLE patients with active disease, MRL-*lpr* mice spontaneously develop splenomegaly and lymphadenopathy. The incidence and severity of lymphadenopathy and splenomegaly and total number of splenocytes were reduced in vaccinated MRL-*lpr* mice compared to control mice (Figures 1D,E).

### Prevnar-13 Vaccination Ameliorates Lupus Dermatitis and Nephritis

**Figure 2:**
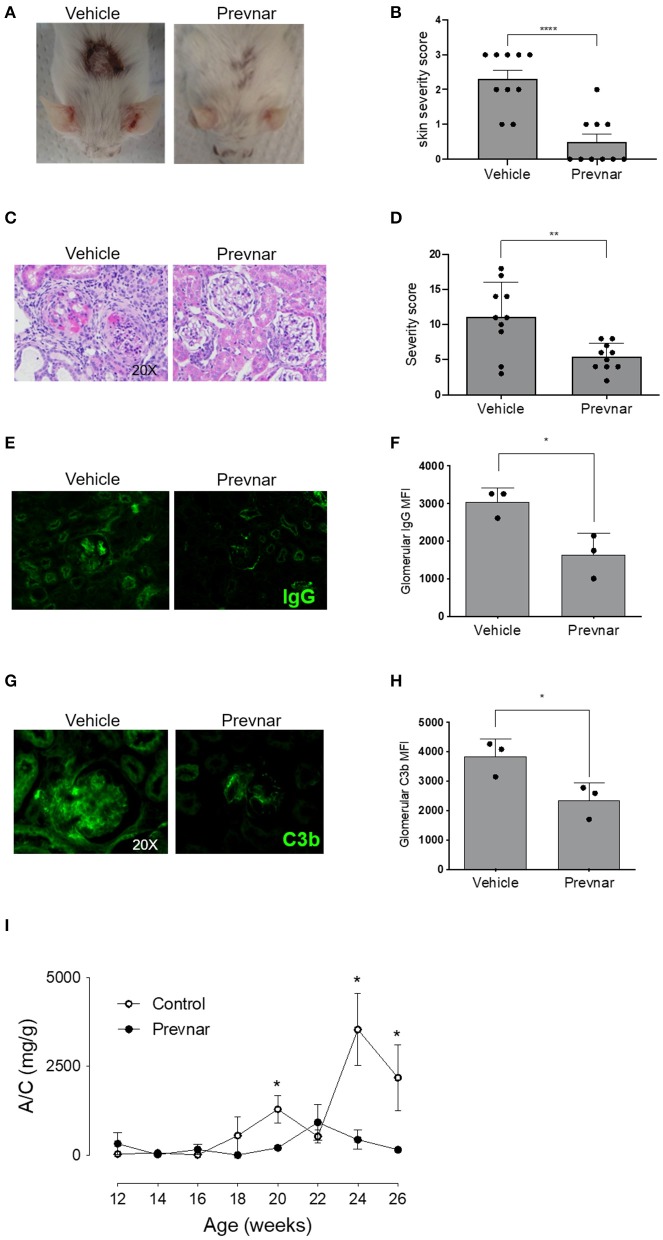
Prevnar-13 vaccination ameliorates lupus dermatitis and nephritis. Representative pictures **(A)** and score **(B)** of skin lesions in Prevnar-13 or vehicle treated MRL-*lpr* mice depicted in Figures 1D,E. Representative glomerular lesions **(C)** and histology severity score **(D)** of nephritis. Representative immunofluorescence staining for IgG **(E)** and C3b **(G)** and data quantification (**F** and **H**, respectively) in the glomeruli of 3 control and 3 Prevnar-13 vaccinated MRL-*lpr* mice (each dot represents the average of glomerular MFI for each mouse). Weekly changes in urinary albumin/creatinine ratio (A/C) **(I)**. Data are presented as mean ± SEM and they are the total of three independent experiments including 3–4 mice per group each. Two mice per group died before 6 months of age; *p < 0.05, **p < 0.001, ****p < 0.0001 by Wilcoxon test **(B,D)**, unpaired *t*-test **(F,H)**, or two-way repeated measures ANOVA test (**I**; comparisons between the two groups at the same time-points).

Worsening disease severity, including cutaneous and renal disease, is the most serious concern preventing widespread vaccination of SLE patients. Cutaneous lupus manifestations in the MRL-*lpr* strain include facial rash, lesions of the back and neck, ulceration and necrosis of the ears. We quantified development of skin lesions in MRL-*lpr* mice vaccinated with Prevnar-13 and in vehicle-treated ones. The incidence and severity of skin lesions were significantly reduced in Prevnar-13 vaccinated mice compared with the control (Figures 2A,B). MRL-*lpr* mice also develop kidney disease characterized by marked glomerular and interstitial inflammation. To evaluate the effect of Prevnar-13 vaccination on kidney disease, we quantified albuminuria and kidney pathology. Similar to its ameliorative effect on skin disease, Prevnar-13 vaccination decreased severity of kidney disease. Vaccinated mice had significantly less inflammatory infiltrates and proliferative glomerular lesions compared to controls (Figures 2C,D and Table 1). Consistently, Prevnar treatment was associated with significantly lower IgG (Figures 2E,F) and C3b (Figures 2G,H) deposition in the glomeruli and reduced albuminuria than in control mice (Figure 2I).

**Table 1:** Single renal histological scores of MRL-*lpr* mice treated with vehicle or Prevnar-13.

	**Vehicle**	**Prevnar-13**
Endocapillary hypercellularity	3 (2.5–3)	1 (1, 2)^*^
Neutrophil infiltration/karyorrhexis	3 (1.5–3)	1 (0.5–1.5)
Fibrinoid necrosis	3 (1–3)	0 (0–1)^*^
Wire loops/hyaline thrombi	1 (0–2)	0 (0–0)
Crescents	2 (1–2.5)	1 (0–1)
Interstitial inflammation	2 (1.5–2)	1 (1–1.5)
Total score	14 (7.5–15.5)	5 (3–6.5)^*^

MRL-lpr mice were treated with vehicle (n = 7) or Prevnar-13 (n = 7) starting at 3 months of age and sacrificed at 6 months of age. A scale from 0 to 3 was used for each lesion, corresponding to the percentage of affected glomeruli (0 = no lesion, 1 = < 25%, 2 = 25% – 50%, 3 = > 50% of glomeruli), based on the International Society of Nephrology/Renal Pathology Society classification for lupus nephritis (30). Values are expressed as median (IQR).

* *p < 0.05 vs. vehicle by Wilcoxon test*.

### Prevnar-13 Vaccination Differentially Regulates T and B Lymphocyte Cytokine Production in MRL-*lpr* Mice

**Figure 3:**
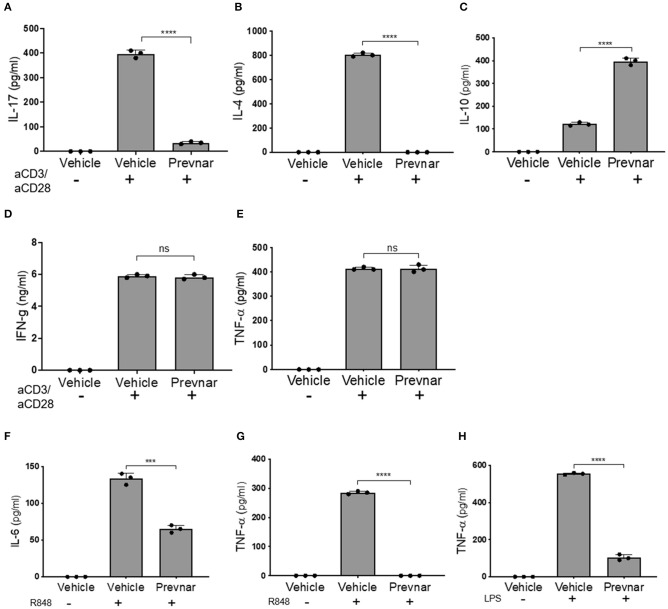
Prevnar-13 vaccination differentially regulates T and B lymphocyte cytokine production in MRL-*lpr* mice. Enriched CD3^+^CD4^+^ T cells isolated from the spleens of control or Prevnar-13 vaccinated MRL-*lpr* mice were stimulated with anti-CD3 and anti-CD28 antibodies for 4 h. Supernatants were harvested to measure cytokine production: IL-17 **(A)**, IL-4 **(B)**, IL-10 **(C)**, and IFN-γ **(D)**, TNF-α **(E)** (ELISA). Splenic B220^+^ B cells were enriched from the same mice and stimulated with the TLR7/8 ligand R848 **(F,G)** or TLR4 ligand LPS **(H)**. At 1 day after T cell stimulation and 5 days after B cell stimulation, we measured IL-6 **(F)** and TNF-α **(G,H)** production in the supernatants (ELISA). Data are presented as mean ± SD and each dot represents the technical replicate of T or B cell responses from 6 mice per group pooled together, ***p < 0.001, ****p < 0.0001, ns, not significant by unpaired *t*-test.

Adaptive immune responses coordinated between T and B cells are essential to SLE pathology. We isolated CD3^+^ T cells and CD19^+^ B cells from spleens of control and Prevnar-13 vaccinated MRL-*lpr* mice to compare their function. Production of IL-17 and IL-4 by anti-CD3/anti-CD28 antibody-stimulated T cell was completely blocked in Prevnar-13 vaccinated mice (Figures 3A,B), while IL-10 production was significantly increased (Figure 3C). IFN-γ and TNF-α production was not affected (Figures 3D,E). We stimulated B cells with two toll like receptor ligands, namely R848 and LPS, that activate TLR7/8 and TLR4, respectively. Similar to T cells, stimulated B cells produced less cytokines (IL-6 and TNF-α) in Prevnar-13 vaccinated mice (Figures 3F–H).

### Prevnar-13 Vaccination Reduces T_**FH**_ and Increases T_**FR**_ Cells in MRL-*lpr* Mice

**Figure 4:**
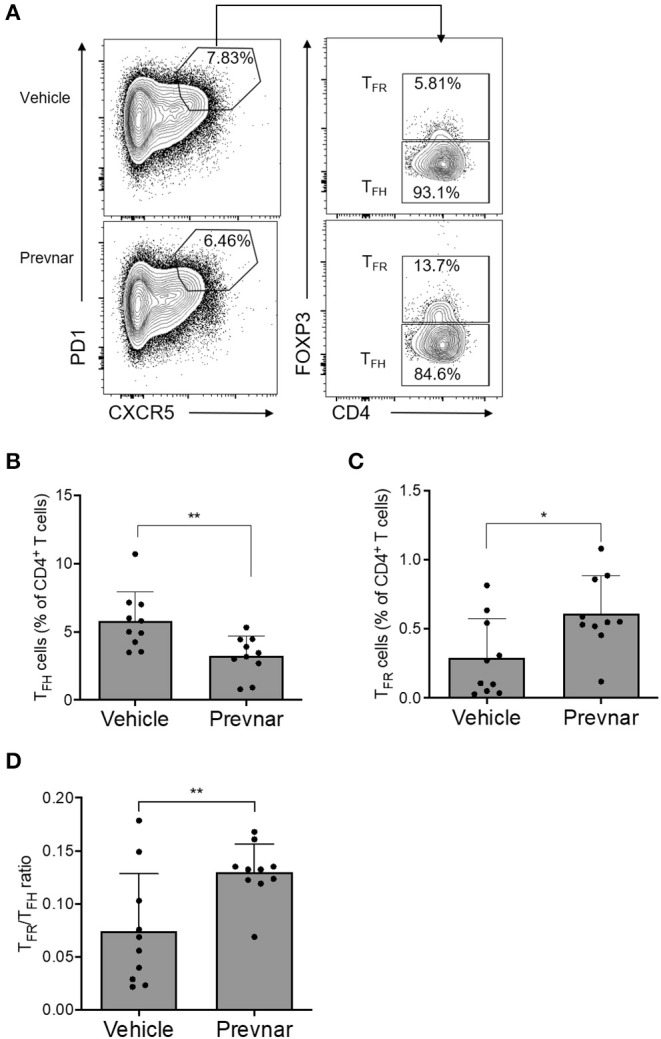
Prevnar-13 vaccination reduces T_FH_ and increases T_FR_ cells in MRL-*lpr* mice. Percentages of CD4^+^CXCR5^+^PD1^+^FOXP3^+^ T_FH_ **(A,B)** and CD4^+^CXCR5^+^PD1^+^FOXP3^−^ T_FR_ **(A,C)** cells, and T_FR_/T_FH_ ratio **(D)** in 6-month old MRL-*lpr* treated with Prevnar-13 or vehicle at 3 months of age. Data are presented as mean ± SD and they are the total of three independent experiments including each 3–4 mice per group, *p < 0.05, **p < 0.001 by unpaired *t*-test.

To understand the mechanisms responsible for amelioration of disease severity associated with Prevnar-13 administration, we measured percentages of total CD4^+^ and CD8^+^ T cells, CD4^+^CD25^+^FOXP3^+^ regulatory T cells (T_REG_), T_FH_, T_FR_, and germinal center B cells (GC B). We found that T_FH_ were significantly lower, while T_FR_ were significantly higher in Prevnar-13 treated mice compared to controls (Figures 4A–C), as was T_FR_/T_FH_ (Figure 4D). No other T cell subsets were significantly different between groups (Supplementary Figure 1), suggesting that the dominant T cell effect responsible for the amelioration of disease severity in Prevnar-13 treated MRL-*lpr* mice is related to changes in T_FH_ and T_FR_ cells.

## Discussion

Our data indicate that anti-pneumococcal vaccine Prevnar-13, effectively produces humoral PPS-specific immunity in lupus-prone MRL-*lpr* mice, while also ameliorating disease severity, a phenomenon associated with reduced T_FH_ cells. Although SLE patients are at increased risk for *Streptococcus pneumoniae* infection (9, 11), vaccination coverage remains dramatically low (32). Limited data are available regarding efficacy of recommended prime-and-boost strategy using Prevnar-13 in SLE patients, but available evidence indicates that they can generate an effective response, albeit inferior compared to healthy controls (11, 33). Our data in MRL-*lpr* mice confirm that Prevnar-13 effectively promotes anti-pneumococcal response, even under MMF immunosuppression, supporting feasibility for vaccination use even in patients receiving immunosuppression.

The effect of Prevnar-13 vaccination on disease severity is poorly defined. Although overall data support its safety, cases of increased disease severity post-vaccination were reported (17, 18, 34). Our data in mice surprisingly showed significant amelioration of both renal and skin lesions in vaccinated mice. Our data were generated in 3-month-old mice, a phase when disease is only at its onset (35). This is rarely the case in humans with SLE whose vaccine indication is related to more advanced disease. It is possible that effects of Prevnar-13 would be diminished or absent in more established/ongoing disease. Another difference between our experiments in mice and human studies is that mice were not receiving immunosuppression, which is rarely the case for SLE patients. However, our data largely support safety of vaccination with Prevnar and support the concept that, if performed early during disease progression, it may be beneficial for disease management.

Generally, polysaccharides are considered to be classic T-cell-independent antigens that do not elicit cell-mediated immune responses. Most bacterial polysaccharides elicit humoral immune responses that result in the induction of low-affinity IgM and some IgG antibodies. Certain polysaccharides of microbial origin have been described to act as potent immunomodulators with specific activity for both T cells and antigen-presenting cells (36). These polysaccharides termed zwitterionic polysaccharides (ZPSs), have both positively and negatively charged moieties and share the same biologic function. These ZPSs include *S. pneumoniae* type 1 polysaccharide (Sp1), *Staphylococcus aureus* type 5 and 8 polysaccharides (CP5 and CP8), and PSA from *B. fragilis*. ZPSs are handled by the MHCII pathway in a manner similar to that documented for traditional protein antigens (8). The first step is uptake of antigen by antigen presenting cells (APCs). Sp1 is endocytosed by professional APCs such as dendritic cells (DCs), B cells, and macrophages.

In an animal model of intra-abdominal surgical adhesion formation, ZPSs (PSA and Sp1) induced formation of a distinct subpopulation of CD4^+^CD45RB^lo^ T cells that produced IL-10 and have anti-inflammatory properties (37). Consistently, we found that Prevnar-13 was associated with increased IL-10 production by T cells which suppresses the IL-2 production required for T-cell clonal expansion (38, 39). Conversely, production of IL-4 and IL-17, two cytokines associated with active disease (40), were inhibited by Prevnar-13.

Polysaccharide-protein conjugates bind to the B cell receptor (BCR) of polysaccharide-specific pre-B cells and are taken into the endosome (41, 42). The protein portion is digested by proteases to release peptide epitopes, which bind to MHCII by replacing self-peptide. Our data show that B cells from Prevnar-13 treated mice display reduced production of TNF-α and IL-6, cytokines that are important for B cell maturation and have been shown to correlate with disease activity (43–45). Whether such effects are due to a direct effect of ZPSs on B cell or through affected T cells modifying B cell activity was not addressed by our studies.

Autoimmune disease in MRL-*lpr* mice is dependent on TLR7, TLR9, and toll-like receptor (TLR) signal transduction molecule Myeloid differentiation primary response 88 (MYD88). Mice lacking either the combined TLR7/9 or MYD88 do not develop autoimmune disease (46). Our *ex vivo* studies demonstrated that B cell from Prevnar-treated mice are hyporesponsive to stimulation with ligands for TLR 7/8 and TLR4, both of which utilize MYD88 for signal transduction. Although pneumococcal polysaccharides alone do not ligate TLRs, the formulation in Prevnar contains TLR2 agonists (47) dependent on MYD88 for signaling (48). While usually viewed in the context of macrophages, endotoxin tolerance causes immune cell changes, including increased regulatory T cells (49). We speculate that Prevnar antigens and duration of their signaling could be producing a relative “tolerant” state compared to untreated mouse, which would be consistent with observed impairments of cytokine production and increased T_FR_ frequencies.

Previous studies indicate that polysaccharide vaccines may generate T_FH_ cell responses in mice and humans, with a link to a functional association with the immunogenicity of the vaccine (50, 51). However, data on the effect of Prevnar-13 on T_FH_ and T_FR_ (a subset of regulatory T cells that controls T_FH_ activation) are missing. Our data show that, in Prevnar-13 treated mice, the T_FR_/T_FH_ ratio significantly increased. How Prevnar promoted these subpopulation changes and their role in modulating disease severity go beyond the scope of the present paper; it is possible to speculate that reduction of peripheral T_FH_ cells is, at least in part, involved in the beneficial effect of Prevnar-13 on disease activity.

In conclusion, our studies showed that anti-pneumococcal vaccination with Prevnar-13 associates with amelioration of disease severity in murine lupus, a phenomenon associated with reduced TNF-α and IL-6 production by B cells and increased T_FR_/T_FH_ ratio. These data provide further rationale and support to current guidelines recommending Prevnar-13 vaccination in SLE patients. Whether these mechanisms extend to other vaccines remains unknown.

## Data Availability Statement

The datasets generated for this study are available on request to the corresponding author.

## Ethics Statement

The animal study was reviewed and approved by Icahn School of Medicine.

## Author Contributions

PC and IT designed the study, with the help from EF, GL, and GZ. CC, CG, SH, and VG performed all the experiments. FS scored kidney lesions. SA performed immunofluorescence staining. PC, JL, and IT wrote the initial draft of the paper. All the authors approved the paper.

### Conflict of Interest

The authors declare that the research was conducted in the absence of any commercial or financial relationships that could be construed as a potential conflict of interest.
