# Supplementary figures and images for: Pneumococcal Polysaccharide Vaccine Ameliorates Murine Lupus
Source: Front Immunol. 2019 Nov 20;10:2695. doi: 10.3389/fimmu.2019.02695 (PMC6879550; doi:10.3389/fimmu.2019.02695)

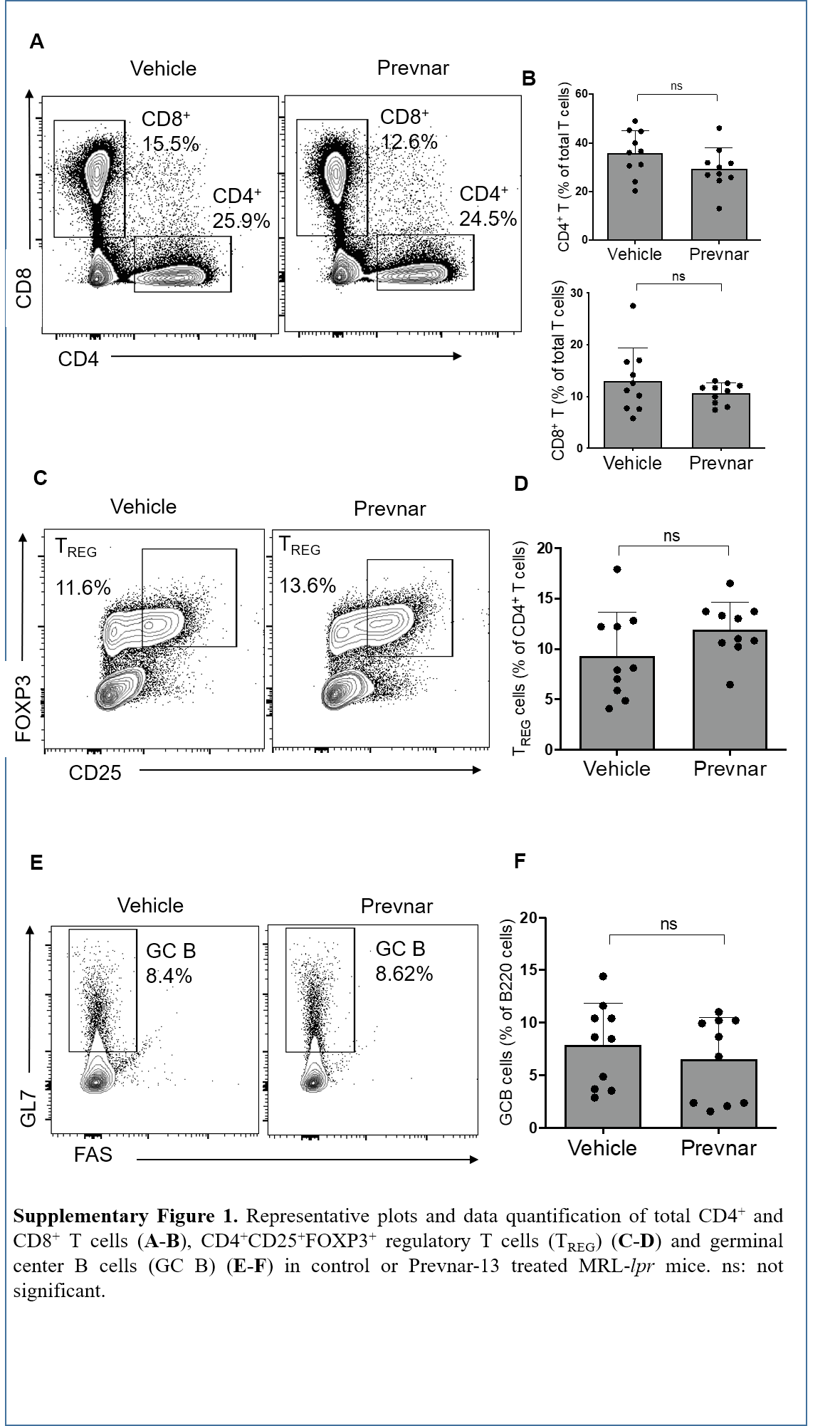

Supplement: Supplementary file 1 [file Image_1.TIF]
